# Ectopic Laryngeal Ossification after Bone Morphogenetic Protein-2

**DOI:** 10.3390/surgeries2040038

**Published:** 2021-11-10

**Authors:** Kirsten Wong, Edward Damrose, Jennifer Long

**Affiliations:** 1 David Geffen School of Medicine, University of California, Los Angeles, CA 90095, USA; 2 Department of Head and Neck Surgery, School of Medicine, Stanford University, Stanford, CA 94305, USA; 3 Department of Head and Neck Surgery, David Geffen School of Medicine at UCLA, Los Angeles, CA 90095, USA; 4 Greater Los Angeles VA Healthcare System, Los Angeles, CA 90073, USA

**Keywords:** larynx, ossification, benign vocal fold lesions, head and neck surgery, swallow/dysphagia, anterior spine surgery

## Abstract

We report two cases of ectopic bone formation in the head and neck following treatment with recombinant human bone morphogenetic protein-2 (rhBMP-2). Surgical pathologic data, laryngoscopy imaging, CT imaging, and patient medical history were obtained. First, we report osseous metaplasia in the vocal fold in a 67-year-old male following mandibular dental implants with rhBMP-2; second, a case of severe bony overgrowth of the larynx and fusion to the anterior cervical spine (ACS) in a 73-year-old male following multiple anterior cervical discectomies and fusions with rhBMP-2. Ectopic bone formation following rhBMP-2 has been previously reported. Adverse events like local swelling and edema leading to dysphagia and even airway obstruction after cervical spine application of rhBMP-2 have also been widely reported. Due to the uncommon nature of abnormal bony growth in soft tissue areas of the head and neck and the previously documented adverse effects of rhBMP-2 use, especially in the cervical spine, we consider the two unusual case presentations of ectopic bony formation highly likely to be linked with rhBMP-2. We urge awareness of the adverse effects caused by rhBMP-2, and urge caution in dosing.

## 1. Introduction

Recombinant human BMP-2 (rhBMP-2) is an osteoinductive growth factor that was FDA-approved in 2002 for treating degenerative disc disease by anterior lumbar interbody fusion (ALIF). It is also currently approved for tibial nonunion and maxillary sinus augmentation [1,2]. While autografts are the first choice for bone repair, rhBMP-2 avoids morbidity and difficulties from iliac crest autograft harvesting. Delivered in an absorbable collagen sponge, rhBMP-2 is used in conjunction with allografts to assist and speed the pace of graft incorporation [3]. Off-label use has expanded to craniofacial surgeries and cervical fusions, particularly in patients with previously failed fusions and increased risk of pseudoarthritis [4,5]. Positive outcomes have been shown in alveolar cleft repair, complex long bone fractures, and free flap mandible reconstruction, but adverse events associated with rhBMP-2 have also been documented in these areas [6,7]. RhBMP-2 is also associated with local swelling that can cause life-threatening airway obstruction [1]. In 2008, the FDA released a public health warning about major complications in cervical spinal fusion with rhBMP-2, highlighting 38 cases of airway and neurological structure compression secondary to severe surrounding edema [8]. Still, the use of rhBMP-2 has continued.

In this report, we introduce two unusual late complications after rhBMP-2 application in the head and neck, both involving laryngeal ectopic bone formation or osseous metaplasia. We also review existing literature regarding adverse events after therapeutic rhBMP-2 application.

## 2. Materials and Methods

The institutional review board considered this retrospective study exempt from review. Patient medical history and clinical data were obtained for both cases through review of the medical records. The patients were both treated by the authors for their laryngological complaints. For case 1, medical history, laryngoscopy images, and surgical pathologic data were obtained. For case 2, medical history and CT scan images were obtained. Operative information was recorded from the surgeon’s operation notes. Expert reads from the clinical pathologist and radiologist were obtained from patient charts. Both patients’ course of treatment and pertinent surgeries were traced and followed to the present time.

## 3. Results

### 3.1. Case Descriptions

#### 3.1.1. Case 1

**Figure 1.**
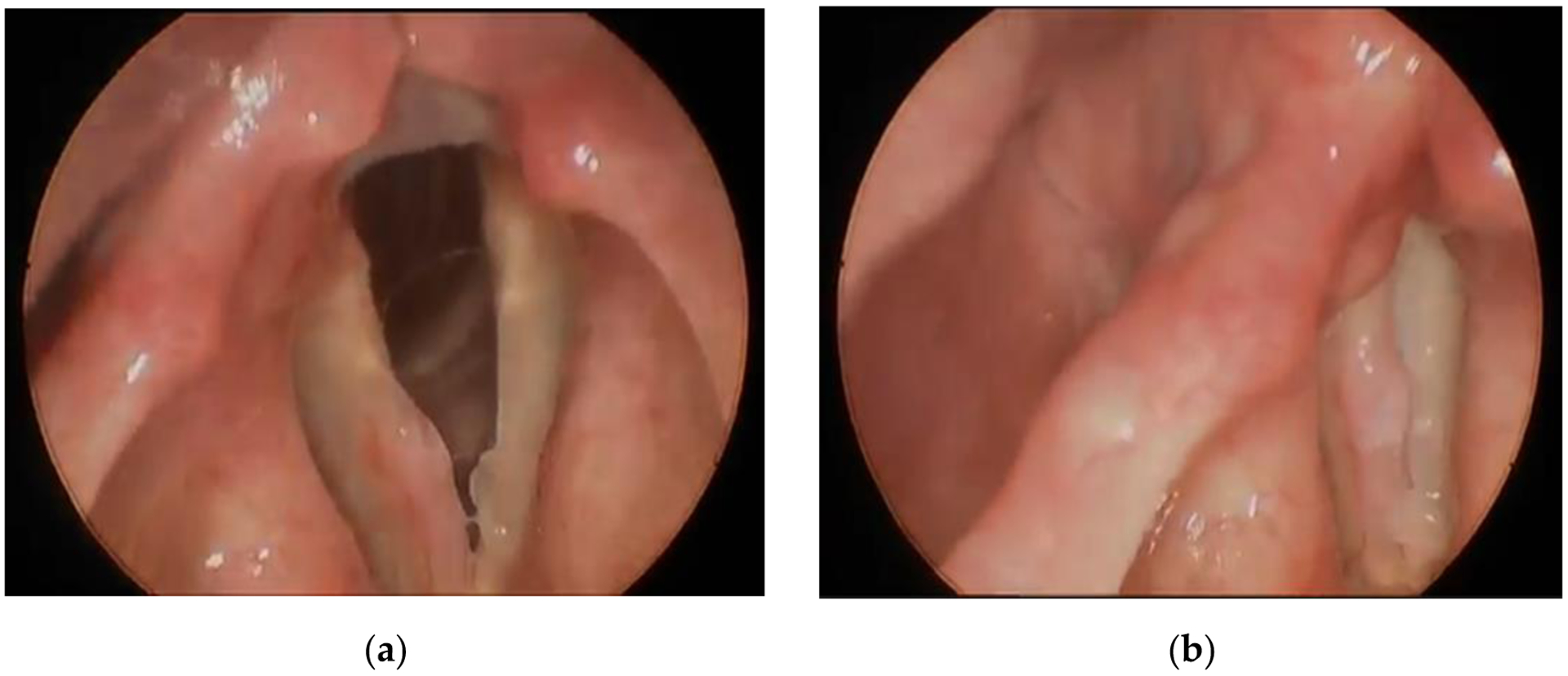
Laryngostroboscopy on presentation shows erythematous right anterior vocal fold thickening: (**a**) open vocal folds; (**b**) closed vocal folds. Figure created by author.

**Figure 2.**
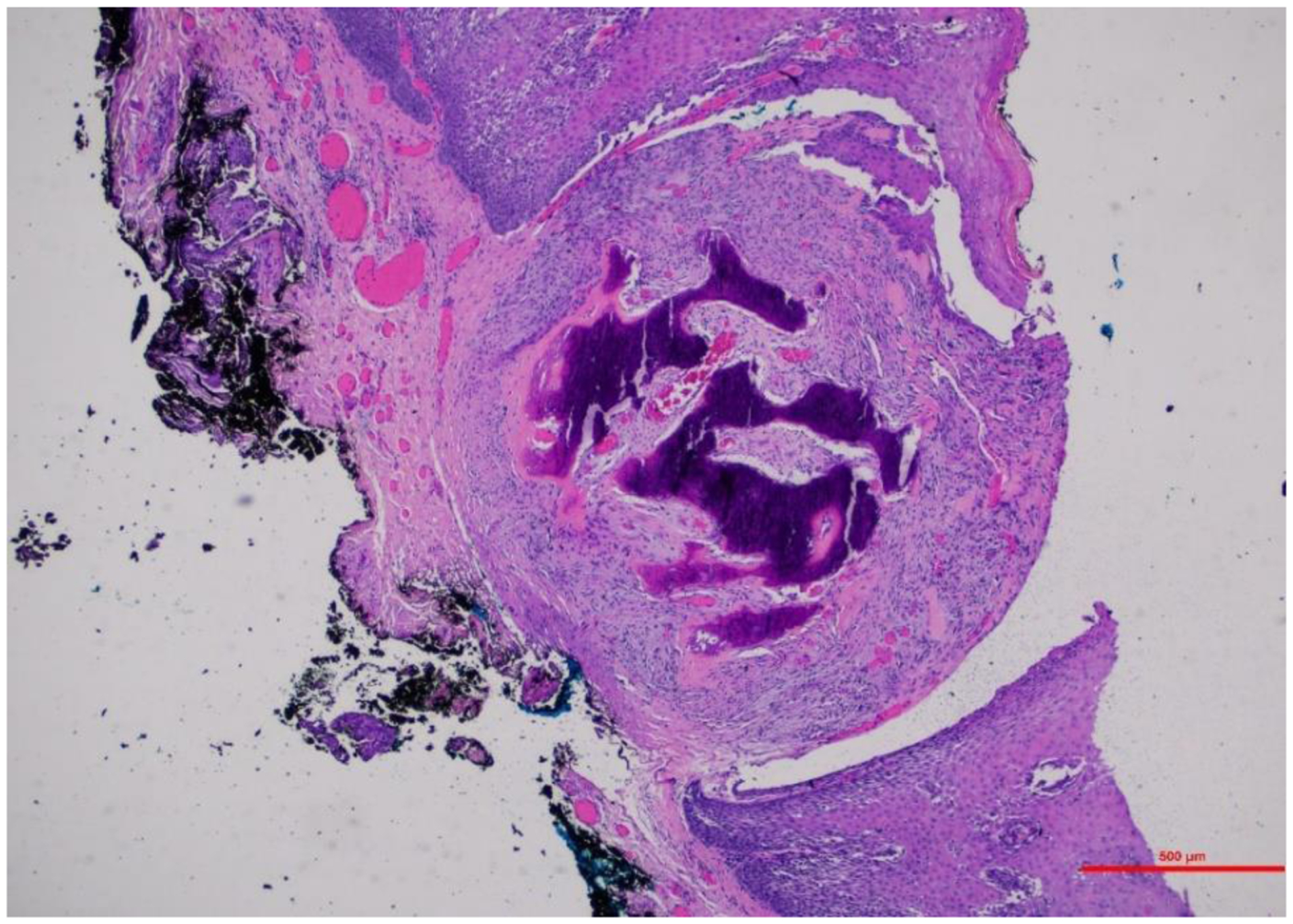
Pathology H&E stain (4×) show hyperplastic mucosa with a superficial, circumscribed focus of metaplastic bone formation.

A 67-year-old male former smoker presented with 3 months of dysphonia with no breathing changes. His history included mandibular dental implants with cadaveric bone graft and rhBMP-2 in 2017, 18 months prior to presentation. Dose information could not be obtained. His history also included lumbar spine surgery without rhBMP-2 and Guillain–Barre syndrome that resolved decades ago. Laryngostroboscopy images at the time of presentation (Figure 1) revealed erythematous right anterior vocal fold thickening and a smaller reactive lesion on the left vocal fold. He was recommended to undergo operative resection for definitive diagnosis. Operative microlaryngoscopy was performed one month after presentation. Findings at surgery confirmed a firm right vocal fold lesion, that was epithelial in origin. CO_2_ laser was used in conjunction with microlaryngeal instruments to remove the vocal fold lesion. The excised tissue was sent for standard pathologic examination (Figure 2). The clinical pathologist described the lesion as “hyperplastic mucosa with a superficial, circumscribed focus of metaplastic bone formation. Surface epithelium was inflamed and exhibited mild atypia in the form of basilar hyperplasia, increased mitotic figures and occasional pleomorphic nuclei.” Post-operatively, the patient’s voice improved but remained rougher than baseline. 18 month follow-up after resection revealed no recurrent disease.

#### 3.1.2. Case 2

**Figure 3.**
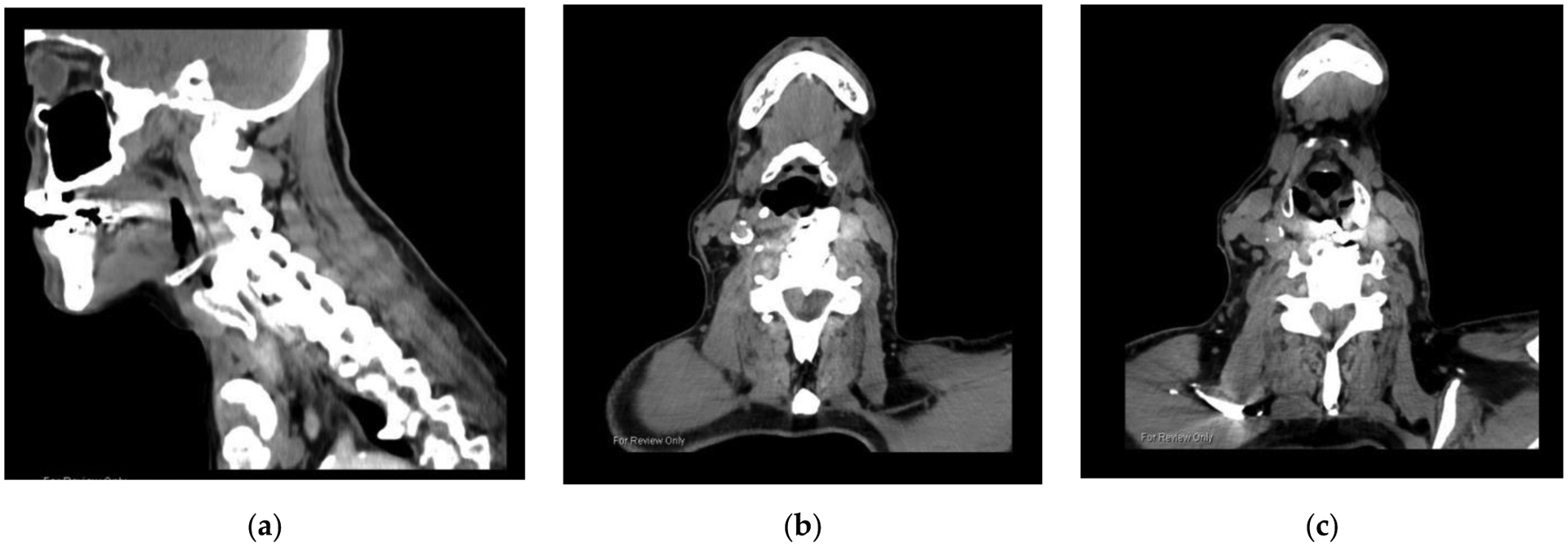
CT images show a large bony overgrowth fusing the larynx to anterior cervical spine: (**a**) sagittal view; (**b**) axial view of upper border at C3 level; (**c**) lower border of bony overgrowth at C6 level. Figure created by author.

A 73-year-old male presented with progressive severe dysphagia for 5 years following 3 separate ACS surgeries with rhBMP-2, culminating in a 4-level fusion. His history included atrial fibrillation, diabetes type II, left-sided weakness following 2 transient ischemic attacks and multiple orthopedic surgeries. He required feeding via gastrostomy tube. During percutaneous endoscopic gastrostomy placement, the patient was found to have exposed hardware in the upper cervical esophagus and low hypopharynx. Cervical spine CT images (Figure 3) revealed bony fusion across levels C3–C6, displacement of the anterior plate, and a large bony overgrowth fusing the larynx to the anterior spine. Given the infection risk of exposed spinal hardware, he was recommended to undergo operative removal of the plate. During that surgery, the left thyroid alae was found to be 2 cm thick, extremely ossified and hypertrophied, and with a bony fusion to the anterior vertebral bodies. Extensive scarring and dense fibrosis involved the superior thyroid artery, thyroid lobe, and strap muscles. The plate was loose and perforated the cervical esophagus. The plate was removed, all bone fusing the larynx and vertebral bodies removed with a drill, and the esophageal perforation repaired primarily and reinforced by the sternocleidomastoid muscle. Posterior cervical stabilization was performed.

## 4. Discussion

In this report, we present two cases of ectopic bone formation involving the larynx that occurred after nearby rhBMP-2 application. Benign bony growths can of course occur in the absence of exogenous BMP. Osteomas affect membranous bones in the paranasal sinuses, external auditory canal, mandible, and other bones in the craniofacial region. Osteomas more commonly occur in divers and swimmers, and inciting mechanisms include persistence of embryonic tissues in adult bone and inflammation from barotrauma, cold temperature, or chronic infection [9,10]. While spine osteomas have been reported, spontaneous osteomas rarely occur outside of the skull and rarely in soft tissue structures like the larynx [11]. The first case of osteoma in the false vocal fold was reported in 2010 [12]. A case of osseous and cartilaginous changes in the larynx was also reported in 2004, but this was a larynx squamous cell carcinoma which differs from the benign environment [13]. In 2017, osseous metaplasia and bone formation was reported in a thyroid follicular adenoma case. While osseous metaplasia is common in malignant neoplasms of the thyroid, it is rare in benign cases [14]. It must also be noted that laryngeal cartilage ossification does normally occur with increasing age, especially in males [15]. However, that normal ossification process is typically symmetrical and only occurs within the actual laryngeal cartilage, not beyond normal cartilage borders Thus, the laryngeal cases we discuss would be highly unusual to occur spontaneously without rhBMP-2 or malignancy.

Regarding the first case, alternative possibilities do exist for the unusual osseous metaplasia development in the vocal fold aside from an aberrant reaction to rhBMP-2. It is possible undiagnosed malignancy was present in the vocal fold or elsewhere. However, malignancy is unlikely because we have followed our patient for over 18 months without evidence of occult malignancy development. This individual had worked as a carpenter and had the additional risk factor of lifelong wood dust inhalation which could have caused irritant laryngitis. His history of Guillain–Barre Syndrome could suggest an autoimmune reaction. However, both these risk factors occur much more commonly in the population than the unusual occurrence of osseus metaplasia in the vocal fold, so we consider them less likely to be causative factors. Although previous reports of vocal fold osseous metaplasia resulting from dental implants have not been reported, the time-relation of his BMP exposure in 2017 and the rare aberrant bone growth that resulted approximately a year later suggest a plausible relationship. Regarding the cervical spine patient in our second case, the degree of laryngeal fusion far exceeds what would normally be considered due to idiopathic vertebral osteoma or age-related laryngeal ossification, so we consider it very highly likely to be linked with rhBMP-2 application.

Previous reports do demonstrate the risks of rhBMP-2 application. A 2010 retrospective cohort study by Yaremchuk et al. identified 260 patients who underwent anterior cervical spine (ACS) procedures with rhBMP-2. This group had significant complications associated with cervical inflammation and soft tissue edema causing higher rates of tracheotomies, unplanned post-surgical intubations, dysphagia or dyspnea, and respiratory failure relative to a control group who underwent the same procedure without rhBMP-2 [16]. Zadegan et al. and Benglis et al. found higher rates of dysphagia or dysphonia, cervical edema, and wound complications in rhBMP-2-treated ACS patients [17,18]. These complications are associated with increased hospital costs and length of stay [4]. Craniofacial surgery applications have been noted to produce primarily edema, erythema, and pain as adverse events [19,20]. In the largest study, Hammoudeh et al. compared 258 rhBMP-2 alveolar bone grafts with 243 traditional iliac crest bone grafts and found significantly more local swelling in the rhBMP-2 group postoperatively [2]. Airway complications were not reported, perhaps because the local edema occurs above the larynx level.

Heterotopic or ectopic bone formation, which can cause spinal compression and unintended fusions, has also been associated with rhBMP-2. In the spine, this is likely caused by leakage of rhBMP-2 from the implant site, with increased risk if the implant is close to the dura mater [1,21]. In 2010, Mroz et al. reported extradiscal and heterotopic bone formation following lumbar rhBMP-2 surgery [22]. Faundez et al. reported ectopic bone formation in off-label uses of rhBMP-2 in posterior and transverse lumbar interbody fusions and anterior cervical fusions [21]. In 2016, Arnold et al. reported higher postoperative heterotopic ossification rates which led to worse outcomes in 224 rhBMP-2 ACS patients compared to 486 allograft patients [23]. Heterotopic ossification in the ACS was also reported by Baskin et al. and Boakye et al. [24,25]. Heterotopic ossification following rhBMP-2 application outside of the spine has also been reported, one case being a rare occurrence of heterotopic ossification in urothelial carcinoma [26].

Adverse events of rhBMP-2 application may be related to their dosing, which lacks consensus and varies by application type and site. Given their short half-lives, BMPs must be present in high enough doses for effective osteoinduction [27]. However, higher rhBMP-2 concentrations are associated with adverse effects like bone subsidence through stimulation of osteoclasts and bone resorption followed by reactivation of new osteoclasts [21]. Since 2007, the FDA recommends a rhBMP-2 concentration of 1.5 mg/mL and total dose of 4.2–12 mg/level for ALIF delivered with absorbable collagen sponges [28]. A 2016 meta-analysis by Hofstetter et al. found ALIF rhBMP-2 dosing varied from 2.1–12.0 mg/spinal level, with complication rates having a positive correlation with dose [29]. RhBMP-2 dosing ranged from 1.4–12.0 mg/level in transforaminal lumbar interbody fusions and 4.2–42.0 mg/level in posterolateral fusions, but complication rates were not correlated with dose in these locations [29]. In anterior cervical disc fusions performed with rhBMP-2, the lowest dose (0.2–0.5 mg/level) had similar fusion rates and lower complication rates compared to surgeries with higher doses (1.1–2.1 mg/level). For posterior cervical fusions, rhBMP-2 doses below 2.1 mg/level were sufficient for optimal fusion rates [29]. In craniofacial surgeries, Hammoudeh et al. used 2.1 mg rhBMP-2 to repair alveolar clefts in children [2]. In maxillary sinus floor augmentation, localized alveolar ridge augmentation, alveolar cleft reconstruction, and cranial defect closures, rhBMP-2 concentrations varied from 0.01–1.5 mg/mL, and post-operative edema was dose-dependent [20]. Other biomaterials for rhBMP-2 delivery aside from the collagen sponge have also been studied, including a demineralized dentin matrix (DDM) which has been shown to be compatible especially for sinus and alveolar ridge augmentation [30]. There is little consensus regarding optimal rhBMP-2 dosing in head and neck surgeries. Interestingly, increased doses may not necessarily increase bone healing but may cause more ectopic bone formation and adverse effects [31]. RhBMP-2 dosing and its relationship to complications should be further researched, as appropriate application can improve clinical outcomes.

Adverse effects of rhBMP-2 use may be explained at the cellular level, where BMP-2 has been shown to induce inflammatory cytokines, activate osteoclasts, and induce adipogenesis and bone cyst formation [1,31]. Briefly, the signaling pathway of the BMP family involves binding to BMP receptors I and II, which are regulated by SMAD activation and MAP kinase to direct transcriptional changes [1,32]. Transcriptional changes induce differentiation of mesenchymal stem cells towards osteoblasts as well as endochondral and intramembranous ossification through regulation of chemotaxis, cell replication and attachment, alkaline phosphatase activity, and osteocalcin mineralization [27,33]. These qualities make rhBMP-2 useful in fracture healing and spine surgeries, particularly those that require bone grafts and in patients with previously failed spinal fusion procedures [3,16]. BMP-2 has also been shown to induce adipogenesis through activation of peroxisome proliferator-activated receptor gamma (PPARγ) signaling, inflammation through cytokines, and osteoclast activity through activation of RANKL [1].

BMP-2 also acts as both a tumor suppressor and oncogenic factor, and endogenous BMP has been shown to be increased in cancers and metaplastic bone formation [3]. Previous studies have associated rhBMP-2 application with increased risk of malignancy in the lumbar spine, but these data are controversial, and no clear link has been proven [1,17]. BMP-2 was shown to promote migration of osteosarcoma cells in vitro possibly by promoting epithelial-mesenchymal transition [34]. In 2001, a rare presentation of osseous metaplasia in nasal polyps was reported, and endogenous BMP-2 expression was shown to be present through immunostaining. Undifferentiated mesenchymal cells in the nasal polyps were proposed to have differentiated into osteoblast progenitors and osteoblasts under the influence of BMPs and TGF-β [35]. A case of intraocular osseous metaplasia in 2005 also revealed endogenous expression of BMP-7, and the ectopic ossification was thought to be aided by local and systemic inflammation [36].

## 5. Conclusions

Based on the two cases presented, and previous evidence of complications reported in the literature, we remind clinicians that rhBMP-2 therapies pose a risk for ectopic bone formation especially in the cervical spine and head and neck region. Additional adverse events like dysphagia, wound complications, and severe edema that can lead to respiratory obstruction are widely reported in the literature. Clinicians should be mindful of the potential for difficult airway management during general anesthesia after rhBMP-2 administration. Patients treated with rhBMP-2 should be closely monitored for complications, and dosing should be carefully considered.
